# Yogurt consumption is associated with a better lifestyle in Brazilian population

**DOI:** 10.1186/s40795-017-0145-3

**Published:** 2017-03-22

**Authors:** Gabriela Possa, José Eduardo Corrente, Mauro Fisberg

**Affiliations:** 1 Department of Pediatrics, Federal University of São Paulo - UNIFESP, Rua Botucatu, 715, Vila Clementino, São Paulo, SP CEP: 04023-062 Brazil; 2 Department of Biostatistics, State University of Sao Paulo – UNESP, Rubião Junior s/no, Rubião Junior, Botucatu, São Paulo CEP: 18618-900 Brazil; 3 Pensi Institute, Sabará Children’s Hospital, São Paulo, Brazil

**Keywords:** Yogurt, Diet, Life style, Cross-sectional studies

## Abstract

**Background:**

The importance of dairy products is recognized for their health benefits. However, additional investigation is required to understand the characteristics of the lifestyle of people who incorporate yogurt into their eating routine. Then, the aim of this study was to compare the lifestyle of yogurt consumers and non-consumers.

**Methods:**

A total of 2610 individuals between the ages of 18 and 59 years were recruited and selected for the study, having as the primary criterion the report of frequency of yogurt consumption. Two study groups were formed: consumers (frequency of yogurt consumption ≥ four times a week during the last year) and non-consumers (consumption frequency of less than once a week), paired for age, sex, and socioeconomic class. A structured questionnaire was applied to obtain the data regarding anthropometric characteristics (weight, height, and waist circumference), lifestyle (food consumption, physical activity, smoking, and alcohol consumption), socioeconomic information (relationship status, level of education, and work) and presence of morbidities. Based on the quantity of consumption in grams/day of yogurt and milk, cheeses, and fruit smoothies, four other analysis groups were formed: LOW-Y/LOW-D (low consumption of yogurt and other dairy products); LOW-Y/HIGH-D (low consumption of yogurt and high consumption of other dairy products); HIGH Y/LOW-D (high consumption of yogurt and low consumption of other dairy products); and HIGH-Y/HIGH-D (high consumption of yogurt and other dairy products). Chi-squared and Student’s *t* tests were used to assess the relationships of these factors.

**Results:**

The yogurt consumers had a higher educational level (≥8 years: 83.8% vs. 79.9%), a higher frequency of individuals working and/or currently studying (67.7% vs. 65.5%), were more physically active at leisure time (17.2% vs. 14.3%), had reduced alcohol intake (3.6 g/day vs. 6.4 g/day) and a lower frequency of smoking (21.7% vs. 25.5%) compared to non-consumers (*p* < 0.05). Besides, individuals included in the Groups HIGH-Y/LOW-D and HIGH-Y/HIGH-D, when compared to those included in the Group LOW-Y/LOW-D, presented a significantly greater intake of calcium, vitamin D, phosphorus, and saturated fat.

**Conclusions:**

This research demonstrated an association between consumption of yogurt and a better lifestyle.

## Background

The importance of dairy products is recognized by scientific and governmental organizations for their health benefits [1–6]. For these reasons, their daily consumption has been encouraged by nutritional guidelines in different countries as part of a healthy diet [5, 7–9]. However, despite the incentives, the consumption of this type of food is low. Specifically regarding the consumption of yogurt, a population-based study conducted in Brazil, which used two non-consecutive food records to evaluate food intake, observed that only 6.2% of the population over 10 years reported the consumption of yogurt in at least one of the two-day food records (Possa et al., data submitted for publication). These data could explain, in part, the high prevalence of inadequate intake, especially of calcium, phosphorus, and vitamins A and D, in Brazilian adolescents, adults and elderly [10–12].

Yogurt, a fermented milk, has a unique nutritional composition compared to milk; it has a lower lactose content (20% to 30% less than milk, making it a good option for individuals with lactose intolerance) and greater vitamin and mineral content, such as riboflavin, vitamin B_12_, calcium, magnesium, potassium, and others, resulting from specific production and fermentation procedures [13–17]. Furthermore, current evidence has shown that yogurt consumption has been associated with a healthier metabolic profile and diet and with reduced risks of weight gain and development of obesity, type 2 diabetes and cardiovascular diseases [14, 18–21]. However, additional investigation is warranted to specifically evaluate the association between yogurt consumption and the typical manner of individuals’ living characteristics, especially those related to their health, such as diet and physical activity.

The aim of this study was to compare the lifestyle of yogurt consumers and non-consumers. The following specific objectives were set: to compare consumers and non-consumers of yogurt with regard to alcohol consumption, smoking status, level of physical activity, nutritional status, presence of morbidities and nutrient intake.

## Methods

### Study design and population

This cross-sectional study included individuals between the ages of 18 and 59 years, from socioeconomic classes A, B, and C, and residents in the urban area of Sao Paulo. Pregnant women and individuals with any physical or mental condition that might make them incapable of participating were excluded from the study.

**Fig. 1:**
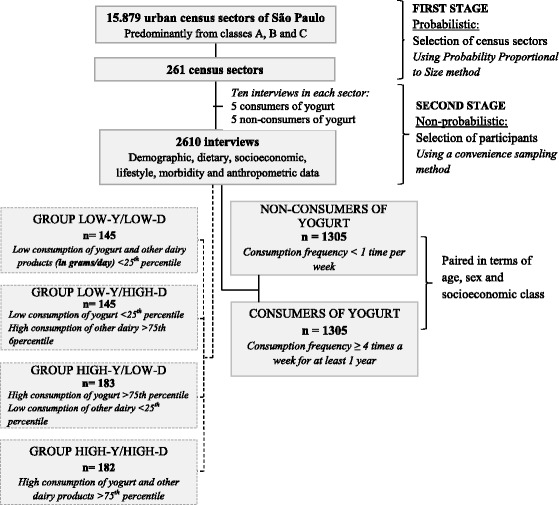
Flow chart of the study São Paulo, Brazil

The sampling process occurred in two stages, the first of which was probabilistic, corresponding to the selection of census sectors, and the second stage was non-probabilistic using a convenience sampling method in reference to the selection of the participants (Fig. 1). In the first stage, of the total of 15,879 census sectors in the urban area of Sao Paulo, with the predominance of socioeconomic classes A, B, and C, 261 censor sectors were selected. This selection was systematically conducted using the probability proportional to size (PPS) method. The second stage was conducted within each censor sector using the proportional quotas relative to the following variables: sex (female, 64%; male, 36%), age (18–29 years, 29%; 30–39 years, 22%; 40–49 years, 25%; 50–59 years, 24%), and socioeconomic class (AB, 43%; C, 57%). The determination of the quotas was based on a study conducted by Possa et al., which aimed to verify the factors associated with yogurt consumption in Sao Paulo [22].

In each sector, 10 interviews were planned, five with yogurt consumers and five with non-consumers, totaling 2610 interviews. The interviews occurred in randomly selected homes using the systematic skip from a home when the interviews were completed.

Yogurt consumers were those who reported a frequency of yogurt consumption ≥ 4 times a week in the last year [23–26]. The group of non-consumers, paired with the consumers for age, sex, and socioeconomic class, consisted of individuals with yogurt consumption frequencies of less than once a week. These pairing variables were selected because they are important confounders, being associated with the consumption of yogurt [22]. The individuals with consumption frequency between one and three times a week, as well as those who reported a consumption ≥ 4 times a week for a period of less than one year were not included in the study.

The project was approved by the Institutional Review Board of the *Universidade Federal de Sao Paulo*. The participants provided their written informed consent.

### Data collection and processing

Data collection was performed by trained interviewers in the participants’ homes during the week and on the weekend.

The interview occurred in two phases, both carried out on the same day. In the first phase, recruiting and selection were performed, and the participants’ demographic, socioeconomic, and yogurt consumption frequency data were collected.

To measure the frequency of yogurt consumption, the participants were initially asked: “Do you usually consume yogurt?” Next, a portfolio was presented containing different brands, versions, and packaging. The objective was to confirm the non-consumption of yogurt by individuals who responded negatively to the initial question and to confirm the consumption of yogurt among those who responded affirmatively. The participants were also questioned regarding the frequency of yogurt consumption (less than once a week; one to three times a week; four or more times a week) and the period of consumption (less than one year; one year or more).

Participants who met the study inclusion criteria continued to the second phase of the interview, in which anthropometric (weight, height, and waist circumference) and lifestyle (food consumption, physical activity, smoking, and alcohol consumption) data were obtained, as well as socioeconomic information (relationship status, level of education, and employment) and the presence of self-reported morbidities, including arterial hypertension, diabetes, cardiovascular diseases, and dyslipidemia.

### Anthropometric data

Anthropometric data were verified in triplicate and according to the procedures recommended by the World Health Organization (WHO) [27]. Weight check was performed using calibrated platform-type digital scales (Wiso®, model W801, capacity for 180 kg and precision of 100 g), and height check was conducted using a portable stadiometer with platform (WCS®, maximal measurement 220 cm, precision of 0.1 cm). The mean values of weight and height were used to calculate the body mass index (BMI), defined as body mass in kilograms divided by the height in squared meters (kg/m^2^). According to the WHO criteria, participants were classified as normal weight, overweight and obese [28]. The abdominal circumference was obtained by measuring the waist at the midpoint between the last rib and the iliac crest using an inextensible metric tape with 0.1-cm precision. To determine the abdominal obesity, the cut-offs proposed by the WHO were used (≥94 cm for men and ≥ 80 cm for women) [28].

### Demographic and socioeconomic data

To determine socioeconomic classes A, B and C, the classification proposed by the Brazilian Association of Market Research Institutes (ABIPEME, 1997), which considers the consumers’ goods and the educational level of the head of the family, was used. This classification divides the individuals into classes A, B, C, D, and E, based on the composite scores: class E (0–19 points); class D (20–34 points); class C (35–58 points); class B (59–88 points); and class A (≥89 points). Class A represents the most favored social stratum, and class E represents the least favored social stratum. It should be emphasized that this classification considers the education level of the head of the family, which will not necessarily be that of the participant.

Furthermore, the individuals were categorized according to their level of education (<8 years of study/≥ 8 years of study), currently working and/or studying (yes/no), relationship status (with a partner/without a partner), and the presence of a child aged 3 to 12 years residing in the home (yes/no).

### Lifestyle data

#### Physical activity

Data on physical activity were collected using the International Physical Activity Questionnaire (IPAQ), long version [29]. The individuals were initially distributed into two categories of physical activity level: sedentary and non-sedentary. For this classification, the four domains of IPAQ (work, means of transport, domestic tasks, and leisure) were considered. Posteriorly, the individuals were classified as active, yes or no, for the “leisure” domain. Physically active was defined as engaging in moderate intensity physical activity at least 30 min a day five days a week or engaging in vigorous intensity physical activity at least 20 min a day three days a week.

#### Consumption of alcoholic beverages and smoking

The consumption of alcoholic beverages was evaluated using the Alcohol Use Disorders Identification Test (AUDIT) [30]. By means of quantitative scores, the AUDIT identifies the low risk use (scores, 0–7), harmful risk use (scores, 8–15), hazardous use (scores, 16–19), and symptoms of alcohol dependence (scores, ≥ 20). The individuals who reported that they did not consume alcoholic beverages did not answer the AUDIT questionnaire.

The participants were classified as current smokers or non-smokers/former smokers.

#### Morbidities

Self-reported morbidity data were obtained by the following question: “Has any physician told you that you currently have any of the following diseases or conditions: hypertension, diabetes, osteoporosis, dyslipidemia, allergy, lactose intolerance, heart disease, obesity, anxiety or depression, Parkinson’s disease, sleep disorder, cancer, rheumatic disease and/or HIV?” Additionally, one should ask these questions: “Are you in treatment for this disease?” “In the past, have you had any of these diseases diagnosed by a physician?” The individuals who responded affirmatively to any of the questions or reported use of medications were considered as presenting morbidity.

#### Food intake data

Food intake data were collected using a Quantitative Food-Frequency Questionnaire (QFFQ) composed of 65 food items with a frequency that varies from 0 (never) to 10 times; units of time that included day, week, and year; and the portion size of small, medium, or large. The median portion is the reference serving size and is presented in household measures and in grams. The food items are organized into the following categories: dairy products including yogurt (natural or with fruits), breads and biscuits, rice and tubers, legumes and eggs, meat and fish, soups and pasta, vegetables, sauces and spices, fruits, beverages and sweets and desserts. An album containing images of domestic tools was used to help complete the QFFQ. The QFFQ was developed and validated for the population of Sao Paulo to evaluate habitual food consumption during the year preceding its application [31].

To calculate habitual intake, the intake frequencies of different items were converted to daily intake frequencies, which were multiplied by the size of the respective portion, obtaining the daily intake of the item. To calculate the energy and nutrient intake, the daily quantity of food consumed was multiplied by the nutritional value of the item obtained from the North American chemical composition table of the Department of Agriculture of the United States (USDA) and the Brazilian Table of Food Composition (TACO) [32]. The nutrients evaluated in the present study were saturated fat, alcohol, added sugar, vitamin D, and the minerals magnesium, phosphorus, and calcium. Added sugar was considered as that added to foods and products during processing or preparation, as well as sugar added to the food at the time of consumption.

#### Quartiles of consumption of yogurt and other dairy products

Based on the quantity of consumption in grams/day of yogurt and milk, cheeses, and fruit smoothies (that use milk), evaluated together here and named “other dairy products,” four other analysis groups were formed, called group LOW-Y/LOW-D (low consumption of yogurt and other dairy products), group LOW-Y/HIGH-D (low consumption of yogurt and high consumption of other dairy products), group HIGH-Y/LOW-D (high consumption of yogurt and low consumption of other dairy products), and group HIGH-Y/HIGH-D (high consumption of yogurt and other dairy products). In this case, the original groups of the study, “consumers of yogurt” and “non-consumers of yogurt” were not considered (Fig. 1).

To structure these new study groups, initially, the values of the 25th and 75th percentiles of yogurt consumption in grams/day were obtained, as well as the values of the 25th and 75th percentiles of consumption of the “other dairy products.” Posteriorly, the consumption characteristics of each group were defined, whereby the consumption of yogurt or other dairy products below the 25th percentile was described as “low consumption” and the consumption of those products above the 75th percentile was described as “high consumption.”

### Statistical analyses

The statistical analyses were performed using Statistical Analysis Software, SAS version 9.2. (SAS Institute, Cary, NC, USA). Statistical significance was set at 5%.

Initially, the percentage of the participants classified as consumers or non-consumers of yogurt was calculated according to their relationship status (with/without a partner); work/study (working, studying/not working/studying); educational level (<8 years/≥ 8 years); child residing in the home (yes/no); consumption of alcoholic beverages (dependent/harmful/risk/low risk); smoking (yes; no/former smoker); sedentary (yes/no); leisure activity (active: yes/no); nutritional status (no excess weight/overweight/obesity); abdominal circumference (adequate/high); and presence of self-reported morbidities, such as hypertension, diabetes, osteoporosis, dyslipidemia, allergy, lactose intolerance, cardiovascular diseases, anxiety or depression, Parkinson’s disease, sleep disorder, cancer, rheumatic disease, and HIV (yes/no). The chi-squared test or Fisher’s exact test was used to compare proportions. Additionally, the consumers and non-consumers of yogurt were compared according to the mean alcohol intake (g/day) using Student’s *t* test. Sex, age and socioeconomic level were not compared between the groups because they were pairing variables.

Finally, the individuals included in the groups LOW-Y/LOW-D, LOW-Y/HIGH-D, HIGH-Y/LOW-D and HIGH-Y/HIGH-D were compared according to the intake of nutrients, such as calcium, vitamin D, phosphorus, magnesium, saturated fat, and added sugar, the variables of age, BMI, abdominal circumference, leisure physical activity (yes/no), sex (female/male), and educational level (<8 years/≥ 8 years). The groups LOW-Y/LOW-D and HIGH-Y/LOW-D were compared to verify the association between the consumption of yogurt and the variables analyzed; the groups LOW-Y/HIGH-D and HIGH-Y/LOW-D were compared to verify whether there were differences between the consumption of yogurt and other dairy products; the groups LOW-Y/LOW-D and LOW-Y/HIGH-D were compared to verify the association between the consumption of other dairy products and the variables analyzed; and the groups LOW-Y/LOW-D and HIGH-Y/HIGH-D were compared to verify the association between the consumption of yogurt and other dairy products and the variables analyzed.

## Results

The socioeconomic and lifestyle characteristics of yogurt consumers and non-consumers are shown in Table 1. Compared to the non-consumers, there were significantly more yogurt consumers who had a higher level of education (≥8 years: 83.8% vs. 79.9%), performed more physical activity in their leisure time (17.2% vs. 14.3%), had a lower intake of alcohol (3.6 g/day vs. 6.4 g/day) and were working and/or studying (67.7% vs. 65.5%) and not smoking (21.7% vs. 25.5%) at the time of the study (*p* < 0.05). However, no statistically significant differences were observed for the following variables: relationship status, child residing in the home, degree of dependence on the consumption of alcoholic beverages, presence of self-reported morbidities, sedentary lifestyle, nutritional status, and high abdominal circumference (*p* > 0.05) (Table 1).

**Table 1:** Socioeconomic and lifestyle characteristics, presence of morbidities and nutritional status of yogurt consumers and non-consumers^a^

Variables	Yogurt consumers (*n* = 1305)		Non Consumers (*n* = 1305)		Total		*p* value
	n	%	n	%	n	%	
Relationship status (with partner)	679	52.0	772	53.7	1451	55.6	0.3883
Work activity (working/studying)*^b^	869	67.7	803	65.5	1672	65.1	0.0058
Education level (≥8 years)*	1093	83.8	1042	79.9	2135	81.8	0.0095
Child at home (yes)	479	63.3	441	66.2	920	35.2	0.1195
Dependence on alcohol^c^							
Dependent	15	2.3	25	3.8	40	3.0	0.3357
Harmful	29	4.5	24	3.6	53	4.0	
Risk	148	22.7	159	24.1	307	23.4	
Low risk	460	70.7	452	68.5	912	69.5	
Alcohol intake (g/day)*^d e^, Mean (DP)	3.6	13.6	6.4	13.7	5,89	7,13	<0.001
Smoking status (currently smoker)*	283	21.7	332	25.5	615	23.6	0.0252
Sedentary (yes)	1138	87.2	1169	89.6	2307	88.4	0.0582
Active in leisure time (yes)*	225	17.2	187	14.3	412	15.8	0.0413
Nutritional Status							
Not overweight	535	41.0	539	41.3	1074	41.1	0.2704
Overweight	420	32.2	449	34.4	869	33.3	
Obese	350	26.8	317	24.3	667	25.6	
Abdominal circumference (adequate)	701	53.7	727	55.7	1428	54.7	0.3066
Hypertension (yes)	189	14.5	203	15.6	392	15.0	0.4479
Diabetes Mellitus (yes)	64	4.0	64	5.2	702	26.9	0.7242
Osteoporosis (yes)	24	1.8	23	1.8	47	1.8	0.8851
Dyslipidemia (yes)	90	6.9	101	7.7	191	7.3	0.4115
Allergy (yes)	94	7.2	109	8.4	203	7.8	0.2754
Lactose Intolerance (yes)	13	1.0	23	1.8	36	1.4	0.0937
Heart disease (yes)	18	1.4	26	2.0	44	1.7	0.2229
Anxiety or depression (yes)	108	8.3	90	6.9	198	7.6	0.1814
Parkinson (yes)	1	0.1	0	0.0	1	0.0	0.4998
Sleep disturbance (yes)	29	2.2	32	2.5	61	2.3	0.6998
Cancer (Yes)	6	0.5	7	0.5	13	0.5	0.7820
Rheumatic disease (yes)	24	1.8	28	2.2	52	2.0	0.5791
HIV (yes)	2	0.2	1	0.1	3	0.1	0.6249

Data was collected 2014 in Brazil

^a^Chi-square test

^b^The total was smaller for this variable than the effective sample due to missing information

^c^Only individuals who reported drinking alcoholic beverages

^d^Student’s *t* test

^e^Adjusting for energy

*p < 0.05

The 25th, 50th and 75th percentile values for the consumption of yogurt were 5.35 g/day, 53.5 g/day and 160.51 g/day, respectively. With regard to the other dairy products (milk, cheeses and smoothies), the values were 85.25 g/day, 194.39 g/day and 338.92 g/day for the 25th, 50th and 75th percentile values, respectively.

Table 2 presents the comparisons between groups LOW-Y/LOW-D and HIGH/Y/LOW-D, and groups LOW-Y/HIGH/-D and HIGH-Y/LOW-D. As expected, in the comparison between groups LOW-Y/LOW-D and HIGH/Y/LOW-D, we noted a significantly greater intake (*p* < 0.05) of calcium, vitamin D, phosphorus, and saturated fat in group HIGH/Y/LOW-D relative to group LOW-Y/LOW-D. The prevalence of women, as well as active individuals in their leisure time, was also greater in group HIGH/Y/LOW-D compared to group LOW-Y/LOW-D (female, 68.3% vs. 56.6%; active leisure, 23.5% vs. 12.4%; *p* < 0.05). For the intake of magnesium and added sugar, and for the variables age, BMI, abdominal circumference, and education level, no significant differences were observed between groups LOW-Y/LOW-D and HIGH/Y/LOW-D (*p* > 0.05). When the comparison was made between groups LOW-Y/HIGH/-D and HIGH/Y/LOW-D, a significant difference was noted in the intake of calcium (1138.90 mg vs. 1052.90 mg), vitamin D (6.75 μg vs. 2.63 μg), phosphorus (1519.12 mg vs. 1466.08 mg), and added sugar (55.79 g vs. 72.02 g) between the groups (*p* < 0.05). However, for the other variables (intake of magnesium and saturated fat, age, BMI, abdominal circumference, physical activity, sex, and educational level), no significant differences were observed between the groups (*p* > 0.05).

**Table 2:** Nutrients intake, demographic, socioeconomic and lifestyle characteristics among study groups

Variable	Group LOW-Y/LOW-D (*n* = 145)		Group HIGH-Y/LOW-D (*n* = 183)		*p* value	Group LOW-Y/HIGH-D (*n* = 145)		Group HIGH-Y/LOW-D (*n* = 183)		*p* value
Mean ± DP^a b^										
Calcium (mg)	495.63	247.57	995.16	247.15	<0.0001	1138.90	308.75	1052.90	308.57	0.0129
Vitamin D (mcg)	1.75	0.60	2.45	0.66	<0.0001	6.75	1.56	2.63	1.62	<0.0001
Phosphorus (mg)	1080.08	192.91	1366.49	192.50	<0.0001	1519.12	230.23	1466.08	230.11	0.0395
Magnesium (mg)	332.19	54.91	336.00	54.92	0.5363	364.73	54.07	360.72	53.97	0.5067
Saturated fat (g)	25.77	6.14	31.75	6.17	<0.0001	35.20	6.14	35.04	6.22	0.8266
Added sugar (g)	60.35	38.17	61.81	29.62	0.7324	55.79	44.67	72.02	44.64	0.0102
Age (years)	40.14	12.19	37.96	12.66	0.1159	40.31	13.01	37.96	12.66	0.0993
BMI (kg/m2)	27.26	5.86	27.02	5.19	0.7049	27.15	6.22	27.02	5.19	0.8388
*Abdominal circumference*										
Women	90.01	14.05	89.47	17.02	0.8251	89.88	16.26	89.47	17.02	0.8652
Men	93.11	15.29	88.63	13.45	0.1030	90.42	13.18	88.63	13.45	0.5002
n and %^c^										
Active in leisure time										
No	127	87.59	140	76.50	0.0104	110	75.86	140	76.50	0.8923
Yes	18	12.41	43	23.50		35	24.14	43	23.50	
*Sex*										
Female	82	56.55	125	68.31	0.0284	94	64.83	125	68.31	0.5066
Male	63	43.45	58	31.69		51	35.17	58	31.69	
Education level										
< 8 years	37	25.52	35	19.13	0.1469	27	25.52	35	19.13	0.9076
≥ 8 years	108	74.48	148	80.87		118	81.38	148	80.87	

Data was collected 2014 in Brazil

^a^Student’s *t* test; ^b^Adjusting for energy; ^c^Chi-square test

Group LOW-Y/LOW-D = low consumption of yogurt and other dairy products (in grams/day)

Group LOW-Y/HIGH-D = low consumption of yogurt and high consumption of other dairy products (in grams/day)

Group HIGH-Y/LOW-D = high consumption of yogurt and low consumption of other dairy products (in grams/day)

The comparison between groups LOW-Y/LOW-D and LOW-Y/HIGH-D and groups LOW-Y/LOW-D and HIGH-Y/HIGH-D is presented in Table 3. The individuals belonging to group LOW-Y/HIGH-D showed significantly higher intakes of calcium, vitamin D, phosphorus, magnesium, and saturated fat and lower intakes of added sugar compared to group LOW-Y/LOW-D (*p* < 0.05). Moreover, these individuals proved to be more physically active than those in group LOW-Y/LOW-D (24.1% vs. 12.5%; *p* < 0.05). Nevertheless, the groups showed no differences with regard to the variables age, BMI, abdominal circumference, sex, and education level. Regarding the comparison between groups LOW-Y/LOW-D and HIGH-Y/HIGH-D, there was a significantly higher intake of all of the nutrients evaluated in group HIGH-Y/HIGH-D, except for added sugar, which was higher in group LOW-Y/LOW-D. Specifically in regard to calcium, the difference between the groups was 1100 mg. The individuals from group HIGH-Y/HIGH-D were predominantly of the female sex (68.7% vs. 56.6%), had a higher educational level (≥8 years: 85.2% vs. 74.5%), and were more active in their leisure time (32.4% vs. 12.4%). Nonetheless, the groups did not differ with respect to the mean age, BMI, and abdominal circumference.

**Table 3:** Nutrients intake, demographic, socioeconomic and lifestyle characteristics among study groups

Variable	Group LOW-Y/LOW-D (*n* = 145)		Group LOW-Y/HIGH-D (*n* = 145)		*p* value	Group LOW-Y/LOW-D (*n* = 145)		Group HIGH-Y/HIGH-D (*n* = 182)		*p* value
Mean ± DP^a b^										
Calcium (mg)	508.80	236.37	1113.20	236.38	<0.0001	602.58	390.03	1709.66	384.89	<0.0001
Vitamin D (mcg)	1.83	1.68	6.63	1.69	<0.0001	2.26	2.89	8.40	2.83	<0.0001
Phosphorus (mg)	1110.60	188.68	1459.18	188.69	<0.0001	1277.67	285.61	1970.07	281.82	<0.0001
Magnesium (mg)	339.92	61.28	348.89	61.29	<0.0001	381.47	62.62	423.60	61.78	<0.0001
Saturated fat (g)	26.87	6.72	33.33	6.72	<0.0001	31.51	7.46	40.24	7.28	<0.0001
Added sugar (g)	63.58	43.71	50.08	43.71	0.0102	72.16	40.46	56.60	39.93	0.0011
Age (years)	40.14	12.19	40.31	13.01	0.9074	40.14	12.19	39.21	12.16	0.4902
BMI (kg/m2)	27.26	5.86	27.15	6.21	0.8829	27.26	5.86	28.28	6.38	0.1602
Abdominal circumference										
Women	90.01	14.05	89.88	16.26	0.9574	90.01	14.05	91.62	14.50	0.4641
Men	93.11	15.30	90.42	13.18	0.3292	93.11	15.30	90.60	15.19	0.3942
n and %^c^										
Active in leisure time										
No	127	87.59	110	75.86	0.0098	127	87.59	123	67.58	<0.0001
Yes	18	12.51	35	24.14		18	12.41	59	32.42	
*Sex*										
Female	82	56.55	94	64.83	0.1491	82	56.55	125	68.68	0.0238
Male	63	43.45	51	35.17		63	43.45	57	31.32	
Education level										
< 8 years	37	25.52	27	18.62	0.1568	37	25.52	27	14.84	0.0156
≥ 8 years	108	74.48	118	81.38		108	74.48	155	85.16	

Data was collected 2014 in Brazil. ^a^Student’s *t* test; ^b^Adjusting for energy; ^c^Chi-square test

Group LOW-Y/LOW-D = low consumption of yogurt and other dairy products (milk, cheeses, and fruit smoothies) (in grams/day)

Group LOW-Y/HIGH-D = low consumption of yogurt and high consumption of other dairy products (in grams/day)

Group HIGH-Y/LOW-D = high consumption of yogurt and low consumption of other dairy products (in grams/day)

Group HIGH-Y/HIGH-D = high consumption of yogurt and other dairy products (in grams/day)

## Discussion

The present study demonstrated that yogurt consumption is associated to a better lifestyle because yogurt consumers have proven to be more physically active in leisure and use less tobacco and alcohol. Additionally, the consumers, compared to the non-consumers, presented a higher education level and a greater frequency of subjects who worked and/or studied at the time of the survey.

The present study identified the differences between the group of yogurt consumers and non-consumers with respect to education and work/study. The importance of the socioeconomic factors for the acquisition of the food groups, especially for those food groups composed of milk and its derivatives, meats, fruits, greens, and vegetables, has already been documented in the literature [33, 34]. Possa et al. investigated the factors associated with yogurt consumption and observed a positive association between the amount of yogurt consumption and *per capita* family income: with increasing *per capita* family income, yogurt consumption increases by 0.61 g [22]. Considering the positive relationship between income and education, it is possible to explain the association observed in this study between the consumption of yogurt and higher levels of education. Additionally, it is likely that the individuals with more education have a greater understanding of nutrition and increased concern regarding their health [35]. However, it is worth noting that the present sample consisted of individuals belonging to the higher socioeconomic classes A, B and C in which the environmental and cultural factors would contribute more toward determining food consumption than in the lower classes.

Several aspects of the present study, e.g., the cross-sectional outline and homogeneity of the groups evaluated, may have hindered us from observing an association between the consumption of yogurt and nutritional status. In the case of morbidities in which no differences were noted between the yogurt consumers and non-consumers, the lack of association could be explained, among other factors, by the low prevalence found for the various conditions assessed, likely due to the study consisting of a younger population (mean age, 39 years), in whom chronic diseases have not yet developed.

Significant variations in lifestyle characteristics, as per consumption of yogurt, are described in other studies, such as the one conducted with Spanish adults, which noted that among those with a frequency of yogurt consumption of more than seven weekly portions, considering the quantity of 125 g as a portion, the level of physical activity was higher and the prevalence of individuals who smoked was lower than that among those with a frequency equal to or less than twice a week (physical activity, 27.1 METs-h/week vs. 20.3 METs-h/week; smoking, 17.8% vs. 27.6%; *p* < 0.05). Additionally, despite a small clinical difference, the BMI values between the groups differed statistically (21.9 kg/m^2^ vs. 22.0 kg/m^2^) (18). Kim (2013) observed that the subjects who never or rarely eat yogurt, compared to those who consume it once or more daily, showed significantly higher values of BMI (23.2 kg/m^2^ vs. 23.9 kg/m^2^; *p* < 0.05) and abdominal circumference (78.9 cm vs. 82.9 cm; *p* < 0.05) [23]. Other authors also observed an association between yogurt consumption and a better metabolic profile, in addition to a protective effect against the development of chronic diseases, such as diabetes, hypertension, obesity, and coronary diseases [19–21]. These aspects could also explain healthier life habits among those who have incorporated yogurt into their routine diet.

To understand the characteristics of yogurt consumers as well as the characteristics of the consumers of other dairy products, additional investigations were performed.

Based on these analyses, we observed a valid association between yogurt consumption and the variable of leisure physical activity and the non-association with the variable of nutritional status. Furthermore, individuals who predominantly made up the group HIGH-Y/LOW-D, characterized by a high consumption of yogurt and a low consumption of other dairy products, were of the female sex, which could be explained by their greater concern with health [36], better knowledge of nutritional themes, and search for nutritional orientation for women [36, 37]. The intake of nutrients, which was the focus of the investigation in these analyses, as expected, was different among those who composed the group HIGH-Y/LOW-D, especially regarding the intake of calcium, which was 500 mg superior, compared with the intake of individuals with a low consumption of yogurt and other dairy products. The higher consumption of other foods rich in calcium by group HIGH-Y/LOW-D, which was not evaluated in this study, could be a likely explanation for this result.

The individuals with a high consumption of other dairy products, but not of yogurt (belonging to the group LOW-Y/HIGH-D), also showed positive characteristics in lifestyle and nutrient intake compared to those with a low consumption of dairy products. This observation led us to consider the likely association between lifestyle factors and dairy products as a whole. Additionally, compared to those individuals with a high consumption of yogurt, but not of other dairy products, group LOW-Y/HIGH-D presented a significantly higher intake of calcium, vitamin D, and phosphorus. Nevertheless, it is important to indicate that group LOW-Y/HIGH-D consisted of individuals with a minimal consumption of 338 g/day of other dairy products and a maximal intake of 5 g/day of yogurt, whereas in group HIGH-Y/LOW-D, we have a minimal consumption of 160 g/day of yogurt and a maximal intake of 85 g/day of other dairy products, that is, a difference in consumption that could explain the results found. In any event, considering the high prevalence of inadequate consumption of nutrients, especially of calcium and vitamin D found in the Brazilian population [10–12, 38, 39], it is important to indicate the role of yogurt as well as other dairy products in providing a greater supply of these nutrients, which could contribute to meet the dietary requirements for Brazilians [15, 40].

The last analysis, which compared individuals with a high consumption of yogurt and other dairy products (HIGH-Y/HIGH-D) and individuals with a low consumption of these foods (LOW-Y/LOW-D) showed interesting results, especially with regard to the intake of vitamins and minerals, reinforcing the importance of this group of foods for the intake of essential nutrients. Additionally, the prevalence of active individuals was 2.6 times higher in group HIGH-Y/HIGH-D relative to group LOW-Y/LOW-D (32.4% vs. 12.4%), which emphasizes the association between the consumption of these foods and a healthier lifestyle. However, regarding the nutritional status, the results remained without significant differences.

The present study has a few limitations. First, it is a cross-sectional study, which does not allow the determination of causality of the events because the exposure and outcome are simultaneously evaluated. Additionally, the second stage of the sampling process was performed in a non-probabilistic manner, that is, by convenience. However, despite these limitations and as far as we know, this is the first Brazilian study that aimed to associate the yogurt consumption and health benefits. Besides this, it is the first study to evaluate the lifestyle of consumers and non-consumers of yogurt using a methodology of recruitment and selection having as a primary criterion the report of frequency of consumption of this food. Two study groups were formed, which were distinct regarding the consumption of yogurt, yet similar regarding the number of members, socioeconomic class, age, and sex. The sampling strategy by pairing in the present study, despite resulting in homogeneous groups, which hinders the observation of some associations among the variables investigated, contributed to eliminating the influence of important confounders, such as in the case of age and sex, thereby preserving the capacity for the generalization of results.

## Conclusions

The study of yogurt has gained importance in the scientific literature in recent years, especially with regard to understanding the relationship between its consumption and the health of the individuals, with interesting results observed. However, the isolated study of one factor may not be sufficient, making it necessary to understand the context in which it appears. Thus, based on the present study, it was possible to understand, in the case of yogurt, the characteristics of the lifestyle of people who, for the most part, incorporate yogurt into their eating routine. The present study warrants additional investigation focusing on the relationship between yogurt consumption and the prevention of chronic diseases.

## Abbreviations

ABIPEME: Brazilian Association of Market Research Institutes
AUDIT: Alcohol Use Disorders Identification Test
BMI: Body mass index
HIGH-Y/LOW-D: High consumption of yogurt and low consumption of other dairy products
IPAC: International Physical Activity Questionnaire
LOW-Y/HIGH-D: Low consumption of yogurt and high consumption of other dairy products
LOW-Y/LOW-D: Low consumption of yogurt and other dairy products
PPS: Probability proportional to size
QFFQ: Quantitative Food-Frequency Questionnaire
TACO: Brazilian Table of Food Composition
USDA: Department of Agriculture of the United States
WHO: World Health Organization
